# Double-Layer Films Based on Furcellaran/Chitosan Complex—Structural and Functional Characteristics of Packaging Materials

**DOI:** 10.3390/ijms262010049

**Published:** 2025-10-15

**Authors:** Ewelina Nowak, Justyna Żak, Magdalena Janik, Agnieszka Cholewa-Wójcik, Lesław Juszczak, Michał Szuwarzyński, Tomasz Mazur, Anna Konieczna-Molenda, Ewelina Jamróz

**Affiliations:** 1 Department of Chemistry, University of Agriculture, ul. Balicka 122, 30-149 Kraków, Poland; justyna08200@gmail.com (J.Ż.); anna.konieczna-molenda@urk.edu.pl (A.K.-M.); ewelina.jamroz@urk.edu.pl (E.J.); 2 NanoLab, University of Agriculture, ul. Balicka 122, 30-149 Kraków, Poland; 3 Department of Product Packaging, Cracow University of Economics, ul. Rakowicka 27, 31-510 Kraków, Poland; cholewaa@uek.krakow.pl; 4 Department of Food Analysis and Evaluation of Food Quality, University of Agriculture, ul. Balicka 122, 30-149 Kraków, Poland; rrjuszcz@cyf-kr.edu.pl; 5 Department of Dietetics and Food Studies, Faculty of Science and Technology, Jan Długosz University in Częstochowa, ul. Armii Krajowej 13/15, 42-200 Częstochowa, Poland; 6 Academic Centre for Materials and Nanotechnology, AGH University of Krakow, al. Mickiewicza 30, 30-059 Kraków, Poland; szuwarzy@agh.edu.pl (M.S.);

**Keywords:** chitosan, furcellaran, double-layer films, polysaccharides

## Abstract

This study involved the creation and characterisation of double-layer films based on furcellaran (FUR) and chitosan (CHIT)—furcellaran complexes. Although chitosan films are quite widely described, a double CHIT-FUR membrane with high solubility and water vapour permeability is seldom reported in the literature. In this work, the physicochemical, mechanical and thermal properties of the obtained double-layer films were examined. The structural properties and morphology of the prepared films were presented using FTIR and AFM analysis. The obtained results confirmed the production of double-layer films, the layers of which differed from each other. The characterisation of the obtained films indicated that the 9:1 ratio (complex_9:1) is superior in terms of uniformity and performance. The obtained double-layer films have the potential to replace traditional plastics in food packaging and may also serve as a new material for medicine capsules in the pharmaceutical industry.

## 1. Introduction

An important element determining the quality of a product and its success on the market is packaging. These materials play an increasingly important role in maintaining the quality of food products. Commonly used food packaging is made of synthetic polymers, due to their low production cost, mechanical resistance, possibility of heat-sealing and flexibility of shape, lightness, barrier properties for gases, as well as excellent transparency and flexibility [1]. However, this type of packaging does not degrade naturally, harming the natural ecosystem and the health of living creatures.

Materials obtained from natural sources, which are also biodegradable materials, have become a popular alternative to the currently used packaging materials. Nonetheless, materials composed of pure biopolymers also have disadvantages and often need to be modified to eliminate problems related to their natural properties [2].

Many researchers around the world are now focusing on developing innovative, multifunctional food packaging that preserves the nutritional value and freshness of the food [3]. At present, there is great interest in materials consisting of several layers (multi-layers) [4]. The ability of polysaccharides to form gels enables research on creating films composed of several layers, in which substances are enclosed that provide active and intelligent properties to materials obtained this way. For example, active agents based on nanomaterials improve the ability of biopolymer films to protect food products from light, moisture, oxygen, and other environmental factors causing food degradation [5]. A number of research initiatives have been undertaken to develop innovative double- and multi-layer packaging films made from biodegradable and compostable materials. Studies on double-layer films [6], double-layer nanocomposites [7], triple-layer packaging materials [8] and also multi-layer materials [9,10] are becoming increasingly common, which significantly improve the barrier and mechanical properties of packaging obtained in this manner. The very promising results obtained by the authors encourage further work on the design and improvement of biodegradable materials obtained via this method.

Naturally occurring polysaccharides, including starch and chitosan, are biodegradable and have documented film-forming properties [6]. When used individually, they have certain limitations with respect to barrier, mechanical and processability properties compared to petroleum-based polymers [11]. The use of techniques, such as biopolymer mixing, structural modifications, and combination with synthetic polymers and/or minerals, has allowed for the expansion of their application by eliminating these technological inconveniences [6,11]. Polysaccharides derived from seaweed, such as alginate, agar, furcellaran, and carrageenan, due to their ability to form gels, their potential for encapsulation, their easy availability, and their biodegradability, are an important source in designing food packaging [12].

Chitosan is a naturally occurring cationic polysaccharide. Presence of free amino groups in the D-glucosamine unit of chitosan is reflected in physical (e.g., solubility), chemical (e.g., reactivity with other groups) and also microbiological properties of this polymer, and makes it unique among polysaccharides [13,14]. It is typically used in biopolymer films due to their high film-forming capacity, biodegradability, as well as antioxidant and antimicrobial activity [15]. Chitosan, due to its antimicrobial and antioxidant properties, is suitable for use in the formulation of active packaging [16]. Chitosan has the potential to greatly interact with components present in food matrices. These feature limits its use in the food industries [13]. Poor mechanical properties, thermal stability and low transparency are still the main factors limiting the use of chitosan [17]. However, the use of chitosan is also restricted due to its low antioxidant activity and insolubility at a neutral pH level. In combination with other polysaccharides and active compounds or their mixtures (fatty acids, lipids, essential oils, flavonoids and various plant extracts) [18], it is a valuable source of packaging films and edible coatings [19]. Complexes of chitosan with anionic polysaccharides can improve the properties of pure polymer and enable the use of chitosan in various applications in the food industry, in particular for the delivery of active compounds, obtaining packaging materials and edible films and coatings.

Furcellaran belongs to the group of natural polymers. It is a sulphated polysaccharide of anionic nature. Furcellaran is structurally and functionally similar to κ-carrageenan, but differs in its number of sulphate esters [20]. This raw material is highly soluble in water, forms gels easily, is biodegradable, biocompatible, and non-toxic. For this reason, it is completely safe and can be consumed by humans [21]. The simplest furcellaran films are made from a mixture of this biopolymer, a plasticiser, and a solvent, which is water. It is also possible to introduce other naturally occurring polysaccharides or proteins, nanoparticles and substances improving physicochemical properties and providing such a composite with active properties. Furcellaran films are also characterised by poor mechanical properties. Depending on the introduced additive, their strength can increase (mixtures with lipids, other biopolymers, nanoparticles and plant extracts) or decrease (mixtures with essential oils) [21]. Due to the ease of gel formation, furcellaran is currently used as a gelling and stabilising agent in food processing [22], as an ingredient for developing edible films and coatings [21] and also multi-layer capsules for drug delivery [23,24].

CHIT-FUR film is innovative because it is made from natural biopolymers, chitosan and furcellaran, which form stable complexes through electrostatic self-assembly. In aqueous solution, upon mixing chitosan with oppositely charged macromolecular polyanions of different types, polyelectrolyte complexes are formed by electrostatic self-assembly [25]. Nevertheless, the investigation of molecular interactions in polysaccharide complexes is difficult because the structure of monosaccharides shows the existence of isomers; their alternating ways of interconnection and the regularity of the monosaccharides are still insufficient [26]. A study conducted by [27], it was proved that combining biopolymers with opposite charges results in the association of flexible chains consisting of macromolecules. As a result of electrostatic interactions, the total charge of the mixture decreased and the formed complex shrinks. In this study, it was verified that obtaining a film from the above-mentioned biopolymers depends on the applied temperature, pH of the environment, ionic strength and the correct proportion between these compounds. According to [28], obtaining transparent and homogeneous furcellaran–chitosan films (CHIT-FUR) is only possible when the appropriate proportions between these polysaccharides are applied. In the cited research, CHIT-FUR complexes were prepared at three different weight ratios of the used polysaccharides (9:1, 8:2 and 7:3). The packaging films obtained in this way were characterised by good mechanical and thermal strength as well as resistance to moisture. Due to their applicative potential, they can be used as packaging materials in the food industry. Herein, our hypothesis was that it is possible to obtain double-layer films based on furcellaran and chitosan/furcellaran complexes, which will have a potential application value in food packaging materials. Additionally, the suitable proportion of polysaccharides in the chitosan/furcellaran complexes will allow selection of a double-layer film with the most interesting structural and functional properties.

Films with a chitosan or furcellaran backbone matrix are the subject of numerous scientific studies. However, at present, to the best of our knowledge, research on the use of chitosan and furcellaran complexes for the synthesis of multi-layer films has been conducted, but only to a limited extent. Yet, chitosan/anionic polysaccharide multilayer films face some constraints, including their poor mechanical properties, high solubility, and barrier properties. Therefore, the aim of this work was to develop double-layer films based on furcellaran and chitosan, based on previously obtained knowledge on the possibility of creating FUR-CHIT complexes [28]. Double-layer furcellaran–chitosan films were fabricated, in which controlled CHIT-FUR ratios (9:1, 8:2, 7:3) facilitated the formation of a functional nanoscale layer that serves as both mechanical reinforcement and a barrier. A high chitosan content (9:1) favoured the development of a dense, integrated network of ionic and hydrogen bonds within the film, thereby slightly improving its physicochemical performance compared to other films.

The first systematic comparison of the structure-performance relationship of CHIT-FUR double-layer films with different proportions was presented. A detailed analysis of the properties of the obtained films, significant from the point of view of packaging, was carried out, based on which the most promising variant of the double-layer film was selected. The conducted research made it possible to obtain a new type of material that can be used in the production of packaging for the food industry.

## 2. Results and Discussion

### 2.1. Scanning Electron Microscopy (SEM)

**Figure 1:**
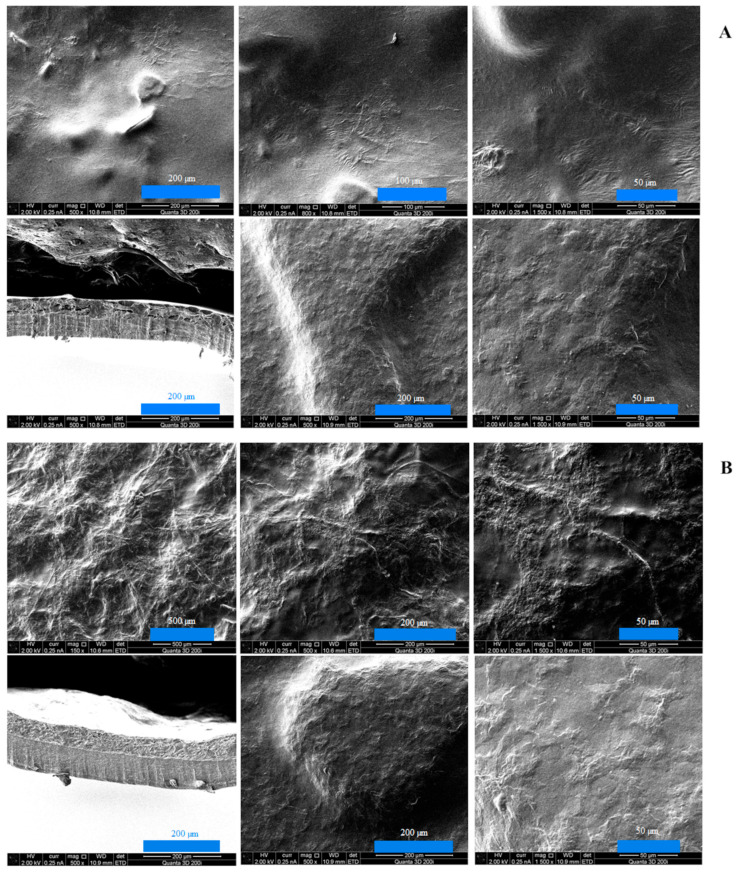

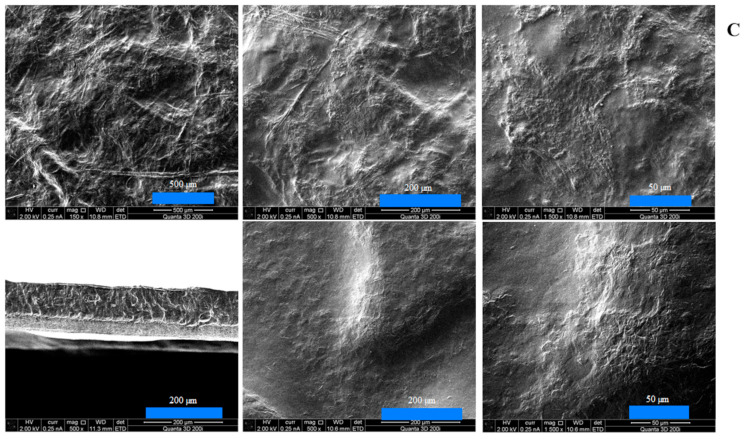
(**A**) SEM images of double-layer films with complex_9:1. The three photos on top—complex layer side (taken at ×200, ×100, ×50 magnifications), two photos on the bottom right—furcellaran layer side (taken at ×200, ×50 magnifications), cross-section—one photo on the left at the bottom (taken at ×200 magnification). (**B**) SEM images of double-layer films with complex_8:2. The three photos on top—complex layer side (taken at ×500, ×200, ×50 magnifications), two photos on the bottom right—furcellaran layer side (taken at ×200, ×50 magnifications), cross-section—one photo on the left at the bottom (taken at ×200 magnification). (**C**) SEM images of double-layer films with complex_7:3. The three photos on top—complex layer side (taken at ×500, ×200, ×50 magnifications), two photos on the bottom right—furcellaran layer side (taken at ×200, ×50 magnifications), cross-section—one photo on the left at the bottom (taken at ×200 magnification).

The results of scanning electron microscopy for double-layer films consisting of a furcellaran layer and a chitosan–furcellaran complex layer at ratios of 9:1, 8:2, and 7:3 are graphically presented in the figure below (Figure 1).

**Figure 2:**
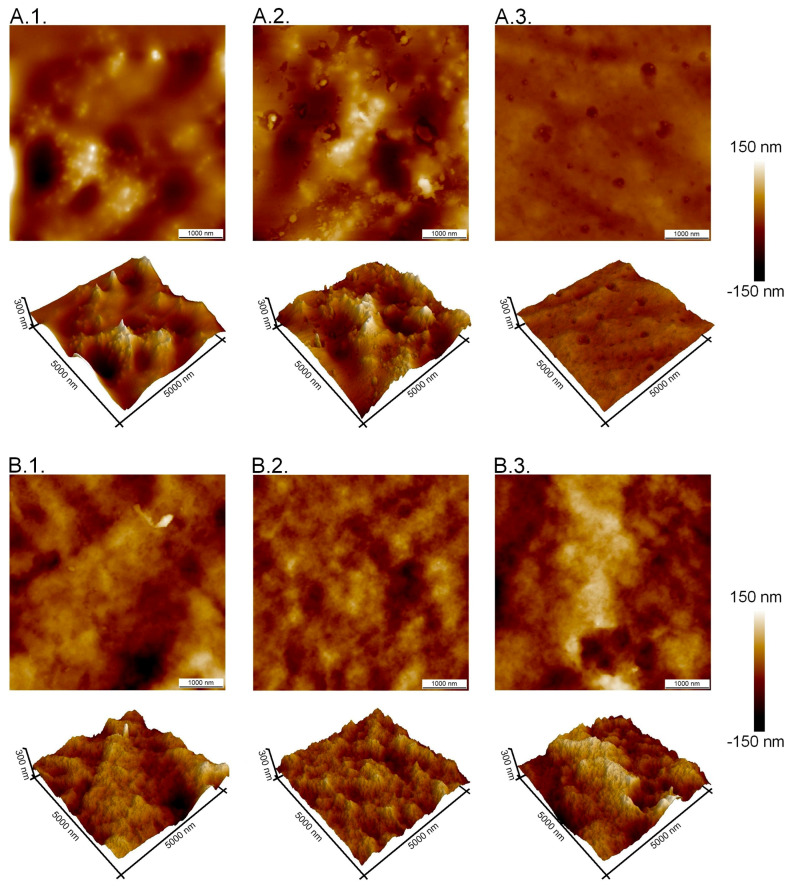
AFM images of double-layer films. (**A.1.**,**B.1.**) means images of double-layer films (CHIT-FUR 7:3 ratio) from the furcellaran layer and the complex layer, respectively. (**A.2.**,**B.2.**) means images of double-layer films (CHIT-FUR 8:2 ratio) from the furcellaran layer and the complex layer, respectively. (**A.3.**,**B.3.**) means images of double-layer films (CHIT-FUR 9:1 ratio) from the furcellaran layer and the complex layer, respectively.

The images obtained using scanning electron microscopy (SEM) for the prepared double-layer films are shown in Figure 1A–C. These images show the surface and interior of the tested materials from the side of the complex (the three photos on top), from the side of the furcellaran layer (two photos on the bottom right) and their cross-section (one photo on the left at the bottom). Analysing the obtained images, it can be seen that the film with the 9:1 complex was characterised by the smoothest surface, which was also confirmed in AFM examination (Figure 2). At this ratio (9:1), the smallest amount of “fluffs” was produced as a result of combining both biopolymers. This can be explained by only a small fraction of the second polymer, which does not disrupt the organisation of the chitosan matrix; thus, the hybrid resembles a structurally pure polymer. However, due to the different chemical character of the two biopolymers—chitosan being cationic and furcellaran anionic—their miscibility is limited. In the remaining composites (complex_8:2 and complex_7:3), the mentioned inhomogeneities were much more numerous, which is consistent with the increased volume of “other” polymer in the mix. The electrostatic imbalance and competition for stabilisation by hydrogen bonds lead to phase separation, and thus more heterogeneous morphology.

At the interface between chitosan and furcellaran layers, a local, multicomponent, partially interpenetrating network is formed, based on (i) electrostatic bridges between protonated –NH_3_^+^ groups of chitosan and sulfonate groups (–SO_3_^−^/S=O) of furcellaran, (ii) a dense network of hydrogen bonds (O–H···O, N–H···O), and (iii) physical entanglement of polymer chains. This results in a prognostic gradient layer, extending from pure furcellaran through an interpenetrating polymer network (IPN) typically tens to several hundred nanometers thick, and further into the chitosan-rich layer.

### 2.2. Atomic Force Microscopy (AFM)

Atomic force microscopy examination results for the double-layer films containing a furcellaran layer and a CHIT–FUR complex layer at the ratios of 7:3 (A.1., B.1.), 8:2 (A.2., B.2.) and 9:1 (A.3., B.3.) are presented in Figure 2. Roughness parameters, RMS, are collected in Table 1.

AFM images show the diversity of the obtained double-layer films. Among the three combinations of prepared chitosan–furcellaran complexes, the 9:1 composite (complex_9:1) was characterised by the most uniform (smooth) surface, which may mean that this ratio of chitosan to furcellaran (9:1) was the most favourable. The RMS surface roughness results showed a significant change in the surface topography structure on the furcelleran side. For samples 7:3 and 8:2, the Rq roughness values were similar and amounted to 24.1 ± 3.2 nm and 27.3 ± 4.6 nm, respectively. For sample complex_9:1, the roughness decreased significantly and amounted to 8.7 ± 0.8 nm. For comparison, the roughness from the complex layer did not change significantly and for each sample had similar values: 28.0 ± 3.4 for complex_7:3 composition, 25.3 ± 3.6 nm for complex_8:2, and 29.4 ± 2.2 nm for 9:1 sample.

The surface of the other two double-layer films (8:2 and 7:3 complexes) was characterised by a less ordered structure, in which a certain number of bulges were observed. First of all, these irregularities were noted for the 8:2 complex. The seen differences in the morphological structure of the surface may be related to interactions between polysaccharides. The differences could also be attributed to the varying water content in the chitosan-furcellaran complexes and the possible interaction of the furcellaran layer with these complexes. This study was also conducted on single-layer coatings obtained from unmodified polysaccharides (chitosan, furcellaran) and their complexes at ratios of 9:1, 8:2 and 7:3 [28]. The obtained topographies showed differences in the surface properties of the analysed films. The material obtained from chitosan was characterised by significant irregularity, which was justified by the presence of crystalline domains. On the other hand, the furcellaran film was characterised by a different, non-porous surface. In the case of the tested composites, the most uniform surface was obtained for the complex at a ratio of 9:1, indicating that, in this combination, the interactions between the atoms of both biopolymers were most optimal. The remaining tested films had regular protrusions, spherical in shape. In these assessments, it was also found that surface dissimilarities may result from the difference in water content. The largest amount of water in the 9:1 complex made the obtained coating smoother and regular. In this work, different results of water content were obtained, and it was found that in the 9:1 complexes, its level was the lowest (8.18%). Nevertheless, the presence of an additional furcellaran layer and possible interactions between furcellaran and the complex may have had an impact on the obtained results. The above-mentioned authors suggest that one possible reason for obtaining different surfaces could be the uneven drying of the film [28]. In films with a high chitosan content (9:1), a more strongly polymerised matrix was obtained, characterised by the dominance of N–H bonds and a higher proportion of protonated NH_3_^+^ groups, which facilitates more effective ionic bridging with the sulfonated groups of furcellaran. At the same time, the furcellaran layer remains sufficiently accessible to allow penetration and the formation of an IPN (Interpenetrating Polymer Network). Films with a higher furcellaran content (8:2, 7:3) contain relatively more anions and fewer accessible amine groups, which results in reduced ionic bridging, increased porosity, and weaker mechanical cohesion between the layers.

### 2.3. Fourier Transform Infrared Spectroscopy

**Figure A1:**
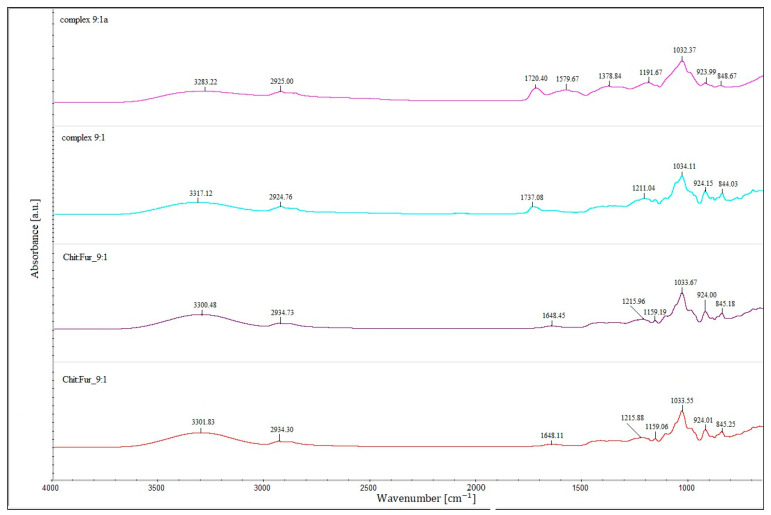
FTIR spectra of CHIT–FUR complexes and double-layer films (9:1).

**Figure A2:**
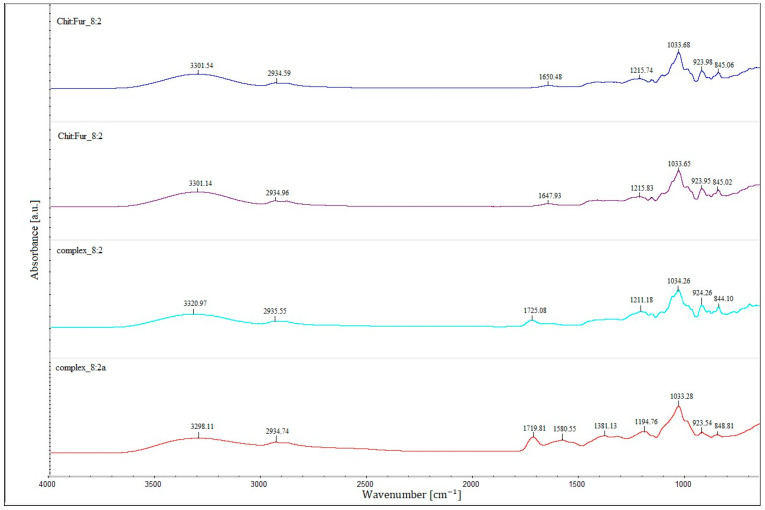
FTIR spectra of CHIT–FUR complexes and double-layer films (8:2).

**Figure A3:**
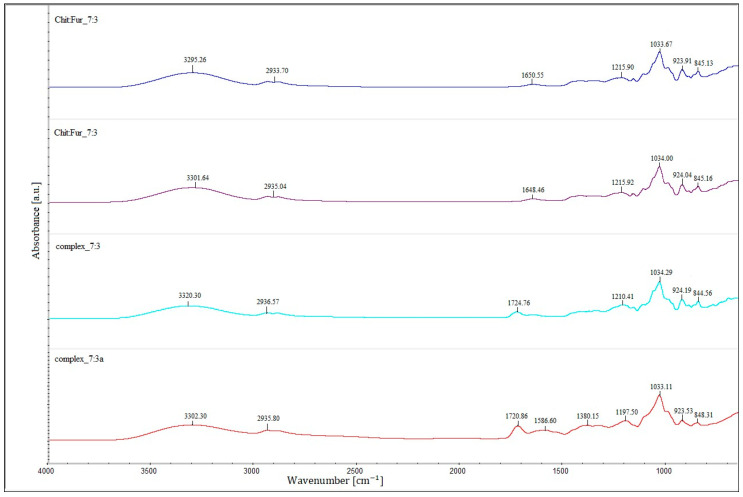
FTIR spectra of CHIT–FUR complexes and double-layer films (7:3).

The Fourier transform infrared spectroscopy results of the films prepared from a single-layer CHIT–FUR complex at ratios of 9:1, 8:2 and 7:3, as well as double-layer films consisting of a furcellaran layer and a layer of the above-mentioned complexes, are presented in Figure A1, Figure A2 and Figure A3 (Appendix A).

The FTIR spectra of CHIT-FUR complexes and double-layer films composed of furcellaran (first layer) and CHIT-FUR complexes (second layer), shown in Figure A1, Figure A2 and Figure A3, had typical bands corresponding to characteristic functional groups of these two polysaccharides. However, the intense band between 950 cm^−1^ and 1100 cm^−1^ that can be observed on each polysaccharide corresponds to the C–C and C=O vibrations of the sugar backbone. The presence of chitosan can be confirmed by the presence of a broad band at ~3300 cm^−1^ assigned to the stretching vibrations of the –NH and hydroxyl (–OH) groups, respectively. The symmetric and asymmetric stretching vibrations of the methylene groups belonging to the pyranose ring were observed at ~2930 cm^−1^. The presence of amide groups (also referred to as I, II, and III amide bands) can be attributed to the bands at approx. 1595 cm^−1^ (stretching vibrations of C=O groups), ~1380 cm^−1^ (bending vibrations of –CH_3_ groups) and ~1650 cm^−1^ (–NH groups). Bands corresponding to the asymmetric vibrations of the –C–O–C– bridges regarding the glucopyranose ring (~1150 cm^−1^ and ~845 cm^−1^) and the =CO backbone stretching (1073 cm^−1^, 1033 cm^−1^) characteristic of polysaccharides were also observed [29]. Due to the presence of free –NH_2_ and –OH groups, which can interact electrostatically or through hydrogen bonding, chitosan can participate in the formation of new connections. The presence of furcellaran, apart from the bands characteristic of polysaccharides, was confirmed by a band at approx. 990 cm^−1^, which is assigned to the –C–O–SO_3_ groups of galactopyranose (containing a sulphate group at C–4carbon). There are also stretching vibrations of the α-D-galactopyranose C–O–C groups (~1150 cm^−1^) as well as asymmetric vibrations of the O=S=O groups and S–O stretching vibrations of sulphate esters, within the range of ~1200 cm^−1^–1450 cm^−1^, respectively [30].

**Figure 3:**
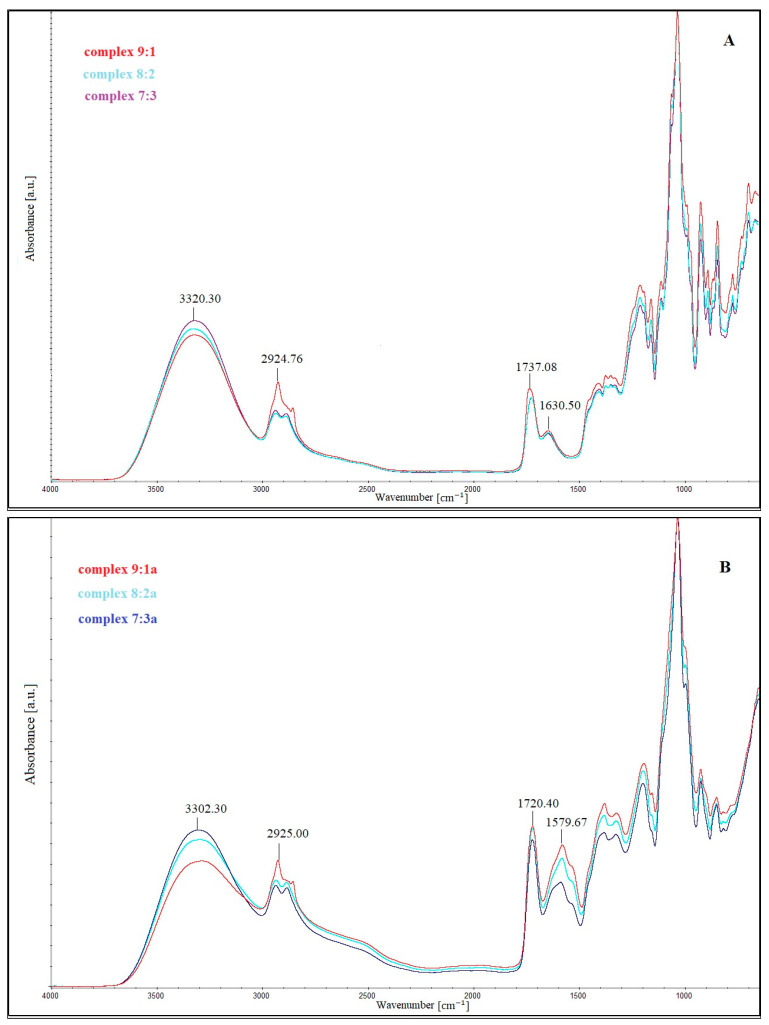
(**A**) FTIR spectra of double-layer films from the furcellaran-chitosan complex layer. (**B**) FTIR spectra of double-layer films from furcellaran layer.

Shifts in the bands arising from the ionised groups of FUR relative to their complex with CHIT indicate intermolecular interactions involving the –OSO_3_^−^ group with the amino group of CHIT (−NH_3_^+^) (Figure 3A,B). The shift in the bands characteristic of chitosan and furcellaran may indicate electrostatic interactions between these polysaccharides forming the complex layer [30]. Specifically, in complexation with CHIT, the peak at ~1580 cm^−1^ of CHIT-FUR was attributed to a symmetric deformation of –NH_3_^+^ groups, suggesting that an electrostatic interaction occurs between ionizable groups of sulphated furcellaran and the amino group of CHIT. The region between 1560 and 1590 cm^−1^ is one of the most important for monitoring interactions between –NH_3_^+^ and –OSO_3_^−^ groups [31,32]. These observations align with the results obtained by the authors [33], who studied the interactions between chitosan and other ionic polysaccharides. Some researchers suggest that the new absorption band around 1410 cm^−1^ is another indication of chitosan and other anionic polysaccharides, especially alginic acid [34,35] and carrageenan [36]. We propose that the superior performance of the CHIT-FUR bilayer arises from the formation of an Asymmetric Ionic–Hydrogen Interpenetrating Network (AIH-IPN) at the interfacial region. This nanoscale IPN is stabilised by electrostatic bridges between protonated chitosan (–NH_3_^+^) and sulphated groups of furcellaran, reinforced by an extensive hydrogen-bond network and partial chain interpenetration.

The double-layer films (designated as the 9:1, 8:2 and 7:3 complexes) shown in Figure 3A,B, composed of a furcellaran layer (measurement from this side marked with the letter a) and a furcellaran-chitosan complex (measurement from their side without the letter a), clearly indicate differences between the layers and between the CHIT-FUR complex (single-layer film). A significant shift in the absorption band from 3300 cm^−1^ to ~3320 cm^−1^ indicates the involvement of amide and hydroxyl groups in the formation of the complex. The analysis of the obtained double-layer films (Figure 3A,B) also indicates a lower intensity of bands in this region (3300–3200 cm^−1^) corresponding to, among others, –OH groups, which may suggest a higher share of hydrogen bondings in the (9:1) double-layer film. An additional band at ~1720 cm^−1^, characteristic of the ester bond carbonyl group, not present in the spectra of pure polysaccharides or the CHIT-FUR single-layer film, may indicate possible interactions between the furcellaran layer and the CHIT-FUR complex (double-layer film). These data correspond to the results obtained from SEM and AFM measurements.

### 2.4. UV-VIS Absorption Spectroscopy

**Figure 4:**
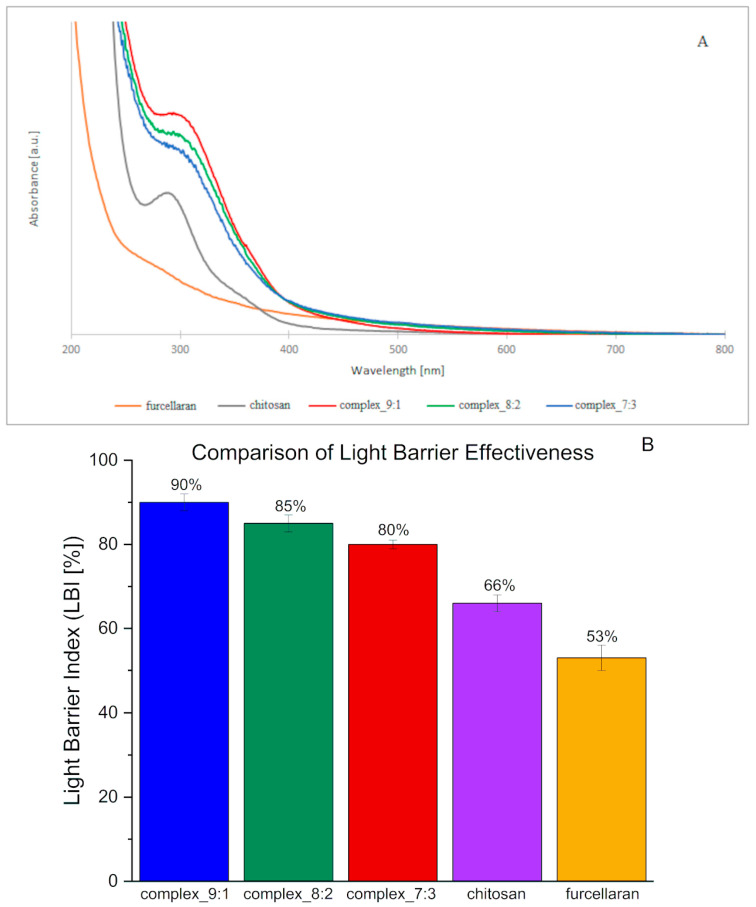
The UV-VIS (**A**) spectra and light barrier effectiveness (**B**) of chitosan, furcellaran, and double-layer films.

The results of UV-VIS absorption spectroscopy concerning the films prepared from double-layer biopolymer matrices are presented in Figure 4A.

Complexes of both of these polysaccharides, as well as the prepared double-layer films, generated spectra very similar to the chitosan spectrum (Figure 4A). A characteristic peak within the range of 270–320 nm was visible on them. The absorption intensity value of single-layer complexes was higher than in the case of films made of basic polysaccharide matrices. The highest absorption intensities were obtained for films containing the 9:1 complex, followed by 8:2 and 7:3. This suggests that with an increase in chitosan content in a given complex, higher absorbance intensity values are obtained. It can also be stated that the higher the furcellaran content, the flatter the peak and shifted towards longer waves (“red shift”). In the study conducted by [37], chitosan-based films were shown to demonstrate good UV barrier properties, which has led to the suggestion that they can delay the oxidation processes of packaged food. The opposite is true for furcellaran coatings, which, according to [38], did not show an absorption peak in the UV-VIS radiation range. The obtained results are consistent with the literature cited above. The addition of furcellaran may weaken the barrier properties of chitosan, as indicated by flattening of the absorption peak.

**Figure A4:**
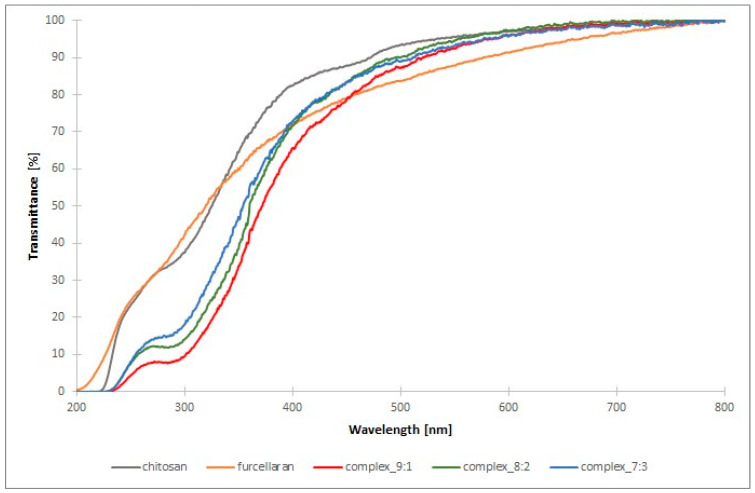
The transmission spectra of chitosan, furcellaran, and double-layer films.

The barrier properties against visible and ultraviolet light of films and packaging materials play a very important role. Transmission spectra in the tested light range (200–800 nm) were performed for pure polysaccharides and their complexes (Figure A4). The transmission spectra revealed that the double-layer films result in an improvement in the barrier properties of the films against UV light, which is reflected by a drastic decrease in the transmittance values of the tested films in the range 200–400 nm with respect to the pure polysaccharides. This effect was observed to a greater extent for the bilayer film with CHIT-FUR complex layer at the ratio of 9:1. The obtained results are in line with a slightly higher value of the quantitative index of light barrier LBI (%) (Figure 4B), which indicates favourable barrier properties. This fact confirms the benefits of using this type of CHIT-FUR complexes for double-layer films, whose purpose is to protect food products from the harmful effects of light.

Double-layer films (9:1, 8:2, 7:3) exhibit a markedly higher light barrier index compared to the individual polymers in their pure form (chitosan and furcellaran). The differences are much greater than the reported uncertainties, indicating that the effect is statistically significant based on the available data. Interactions between anionic furcellaran and cationic chitosan lead to the formation of networks and complexes that enhance light absorption and scattering, thereby reducing transmission. A similar trend has been described in the literature: furcellaran–chitosan composites (especially with additives) demonstrate superior UV-barrier properties compared to the single components [39]. The slightly higher LBI value, and thus the more effective UV barrier, is observed for the film with a higher chitosan content, suggesting that in this case, a denser and more compact network with an enhanced light scattering capacity was formed. In contrast, when the furcellaran content increases, the film structure becomes more homogeneous and transparent, and therefore less effective in blocking light.

### 2.5. X-Ray Diffraction (XRD)

**Figure 5:**
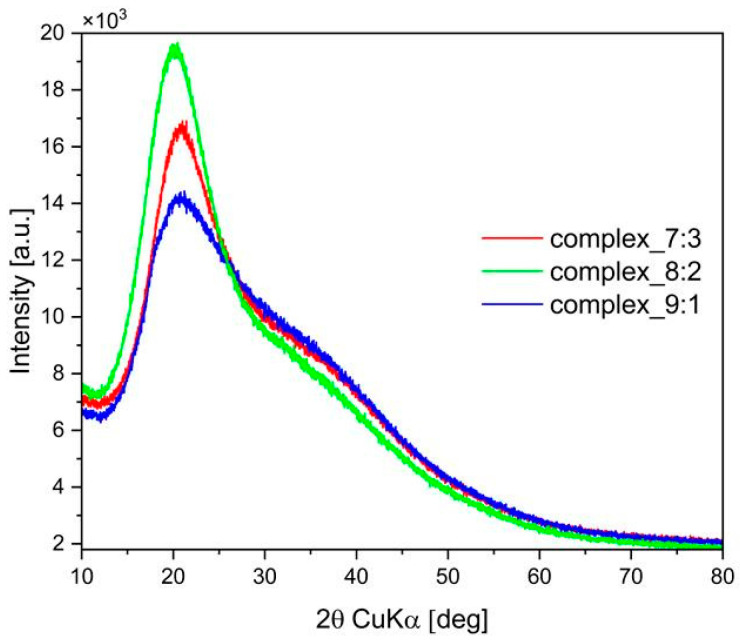
XRD patterns of the double-layer films.

The X-ray diffraction patterns of the double-layer films are presented in Figure 5.

The diffractograms of the films reflect the amorphous structure of the polymers from which they are composed (Figure 5). A Bragg reflection was observed at approximately 2Θ = 20°, which is characteristic of furcellaran. The presence of chitosan in the films caused a shift toward higher angles, 2Θ = 21–22° (2Θ = 21° for 7:3 and 2Θ = 22° for 8:2 and 9:1), which may indicate a compact crystalline structure of the inner layer. The broadening of the XRD peak also suggests a partially crystalline or less ordered structure in the case of the complex_9:1. The complex_8:2 shows a greater number and arrangement of crystallites, which correlates with the higher thermal stability revealed by TGA studies. The complex_9:1 also demonstrated high stability, as evidenced by the highest ΔH (statistically significant) and the most favourable mechanical properties. However, it contains the smallest amount of furcellaran, which means it does not achieve such a favourable inner layer with a high number of electrostatic interactions between –SO_3_^−^ and –NH_3_^+^.

### 2.6. Film Thickness, Water Content and Solubility of the Film

The film thickness, water content and solubility results of the double-layer films consisting of a furcellaran layer and a CHIT–FUR complex layer at the ratios of 9:1, 8:2 and 7:3 are presented in Table 2. The thickness values of the prepared double-layer films were similar. Therefore, it could be concluded that despite the use of different chitosan-furcellaran ratios, this parameter did not change and remained constant. These observations were in line with results described in article [28], where the introduction of furcellaran into the chitosan matrix did not cause changes in the film thickness, and the obtained results were also comparable.

Water content is a significant parameter characterising packaging materials, especially those intended for food contact. It influences the product’s shelf life during both storage and processing. The water content of the tested double-layer films rose with an increase in the share of furcellaran in the complex. The performed statistical analysis showed that the coating containing chitosan and furcellaran at a ratio of 9:1 statistically differed from the other samples. The other tested materials did not show statistically significant differences. The highest solubility, amounting to 63.51%, was characterised by the film with the addition of the complex at a ratio of 9:1. However, all the obtained results for this parameter were similar to each other and did not differ statistically. Such a high solubility results in some limitations in packaging materials but may be beneficial, for example, in the encapsulation process or edible coating materials. The relatively high water solubility of the obtained chitosan–furcellaran films makes them excellent candidates for applications requiring rapid release of embedded substances (e.g., soluble sachets, active layers). However, this property prevents their use as long-term barriers in humid environments [40].

According to [28], the water content in furcellaran films (24.62%) was almost twice as high as in the case of chitosan coatings (14.96%). The value of this parameter also increased as a result of combining both biopolymers with each other. The film made of the 9:1 complex was characterised by the highest water content (30.85%), while the values for the 8:2 and 7:3 complexes were comparable (21.17%, 22.32%). The introduction of furcellaran, which contains sulphate groups, increased the hydrophilicity of the material, making it easier to combine with water. In the above-mentioned research, the solubility of the same materials was also measured. It was proven that furcellaran coatings are completely soluble in water, while chitosan is soluble in 48.67% water. In the case of films made from chitosan-furcellaran complexes, the lowest water resistance was observed in the film with a 7:3 ratio (66.08%), followed by the 9:1 ratio (52.22%) and the 8:2 ratio (39.33%), which proved to be the least soluble. This behaviour was explained by the lower access to hydrophilic groups, resulting from the formation of a compact system. The lower solubility of chitosan films resulted from their more rigid structure and stronger crystallisation. The addition of furcellaran significantly increased this parameter. This was due to the increased availability of hydrophilic groups, which is not beneficial in the case of packaging products with a high humidity level.

The average results of the contact angle measurement for double-layer films consisting of a furcellaran layer and a CHIT–FUR complex layer at the ratios of 9:1, 8:2 and 7:3 are presented in Table 2. Analysing the obtained results of the tested films, it can be stated that the contact angle was higher in the case of the material from the side of the CHIT–FUR complex layer (“top”). In the case of the other furcellaran side (“bottom”), the obtained values were lower. The slightly higher contact angle values, concerning both sides, were found for the double-layer film with the complex having the ratio of 9:1. With an increase in the furcellaran content, the values of the tested parameter decreased. The exception was the film with a 7:3 ratio, the value of which was slightly higher for the “top” layer of the material than that obtained for the 8:2 film, but they were, nonetheless, comparable. The contact angles of the tested films were less than 90°, which indicates their hydrophilic nature. This study was also conducted for single-layer films composed of furcellaran, chitosan, and their complexes at ratios of 9:1, 8:2, and 7:3 [28]. It was shown that the highest contact angle value was characteristic of the chitosan film (90.16°), while the lowest value was observed in the furcellaran film (55.50°). In the case of the material prepared from a complex of both biopolymers, the value of the tested parameter decreased along with the increase in the share of furcellaran. Films made of this polysaccharide generated a contact angle of less than 90°, which is why they were considered hydrophilic. This behaviour was caused by the presence of functional groups (hydroxyl and sulphate) on the surface of the coating. The opposite was the case with chitosan films, whose contact angle was greater than 90°. That is why they were classified as a hydrophobic material generated as a result of the presence of, among others, amine groups. Increasing the share of furcellaran in the complex of both biopolymers caused a decrease in the water contact angle, as a result of which the tested material became more hydrophilic [28].

### 2.7. Water Vapour Transmission Rate

**Figure 6:**
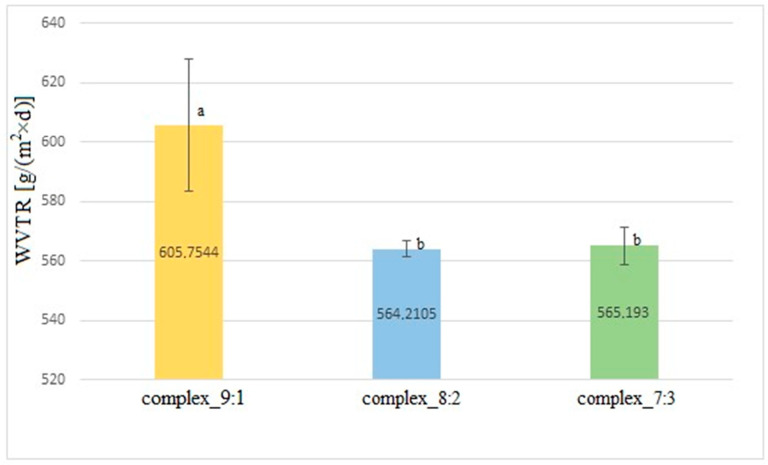
Average water vapour transmission rate through double-layer films.

The average results of the water vapour transmission rate measurements through double-layer films consisting of a furcellaran layer and a CHIT–FUR complex layer at the ratios of 9:1, 8:2, and 7:3 are presented in Figure 6.

The highest water vapour transmission rate (WVTR) was observed for the double-layer film with the complex having the ratio of 9:1, while for the other films (8:2 and 7:3), comparable results were obtained. After statistical analysis of the results, it was found that the complex_9:1 sample differs statistically from the others, while the complex_8:2 and complex_7:3 films do not show any significant differences in comparison to one another. In the trial conducted by [28], the highest WVTR value (approx. 900 g/m^2^·d) was characteristic of the chitosan film, while the lowest (600 g/m^2^·d) was typical of the furcellaran film. Materials from complexes of both of these compounds at the ratios of 9:1, 8:2 and 7:3 were characterised by an intermediate water vapour transmission rate. It was verified that the introduction of furcellaran into the chitosan matrix caused a decrease in the WVTR parameter. This was instigated by the thermal treatment of furcellaran, which lead to the decomposition of this compound and degradation of its chain, which could have resulted in a weakening of the interactions between the implemented biopolymers. These examinations allowed us to confirm the highest WVTR value obtained for the film with the highest share of chitosan.

### 2.8. Colour Measurement Parameters

The average colour measurement results of the double-layer chitosan–furcellaran films consisting of a base furcellaran layer and a layer comprising the complex of the above-mentioned polysaccharides at the ratios of 9:1, 8:2 and 7:3 are presented in Table 3.

Along with the increase in furcellaran content, the brightness (L*) and whiteness index (WI) of the tested films increased. The samples obtained negative values of the a* parameter, which indicated that the green colour was involved, which decreased for the film with the 7:3 complex. The b* coordinate value increased with the increase in the chitosan content. This biopolymer is characterised by a natural, yellow colour, which is why the films containing its highest ratio are characterised by a more yellow shade. The calculated total colour difference indicated that the tested samples differed significantly from the white background, which was easy to observe with the “naked eye”. The same results were obtained in the research conducted by [28], where it was observed that single-layer films made of furcellaran, chitosan, and their complexes at the ratios of 9:1, 8:2, and 7:3 were transparent and slightly yellow. This was confirmed by high values of the L* parameter, negative values of the a* coordinate, and positive values of the b* coordinate, which indicated high brightness of the coatings, as well as a predominance of green-yellow colour.

### 2.9. Mechanical and Thermal Properties of the Tested Materials

**Figure A5:**
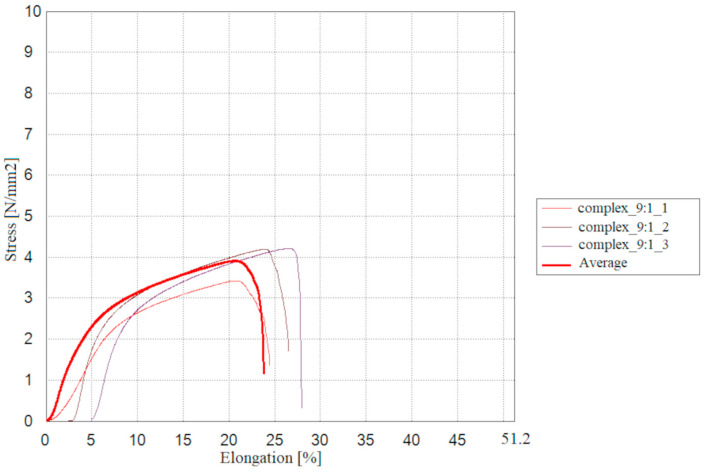
Stress–strain (elongation) curve of double-layer films (9:1). The curves represent three independent experiments (complex_9:1_1, complex_9:1_2 and complex_9:1_3) and the average curve.

**Figure A6:**
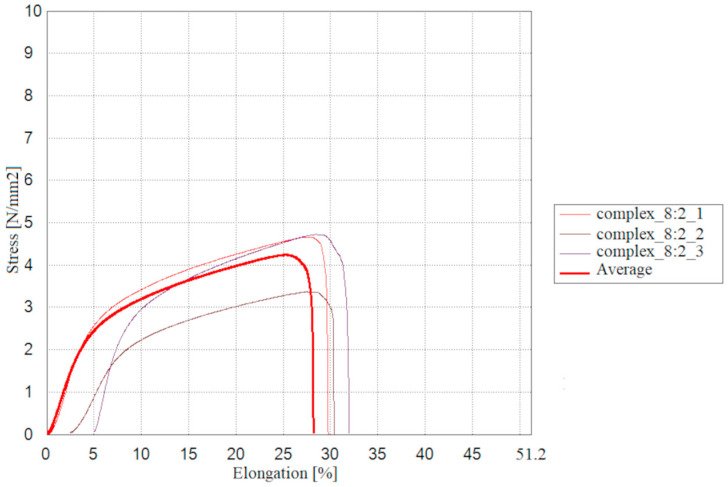
Stress–strain (elongation) curve of double-layer films (8:2). The curves represent three independent experiments (complex_8:2_1, complex_8:2_2 and complex_8:2_3) and the average curve.

**Figure A7:**
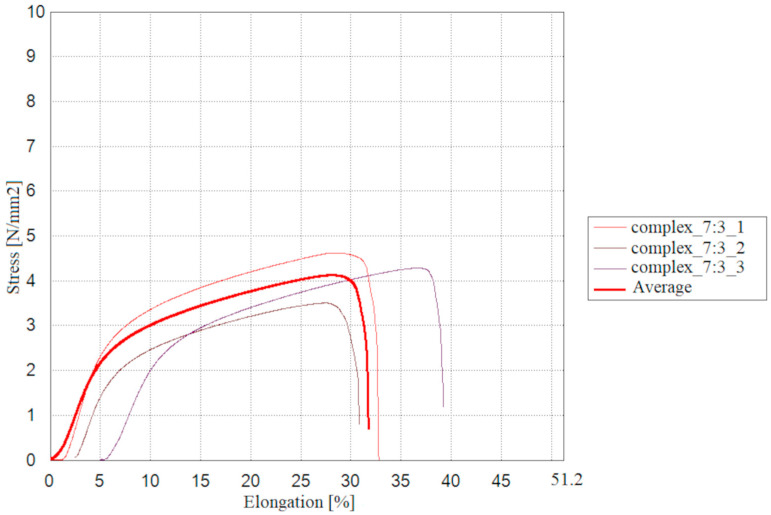
Stress–strain (elongation) curve of double-layer films (7:3). The curves represent three independent experiments (complex_7:3_1, complex_7:3_2 and complex_7:3_3) and the average curve.

The results of measurements for the mechanical properties of the double-layer films consisting of a furcellaran layer and a chitosan-furcellaran complex layer at the ratios of 9:1, 8:2 and 7:3 are presented in Figure A5, Figure A6 and Figure A7 and Table 4.

The conducted tests allowed evaluation of the prepared double-layer films in terms of their mechanical properties. The film containing the complex at the ratio of 8:2 exhibited slightly higher values of maximum breaking load, tensile strength, stretch index, and breaking length, suggesting a positive trend in resistance to destructive forces. The complex_7:3 film, on the other hand, reached breaking length more quickly, but showed the highest maximum elongation and the lowest elastic modulus, indicating that this coating is more flexible and easier to deform. Statistical analysis revealed significant differences were observed only in the maximum elongation test, where the complex_9:1 and complex_7:3 films differed from each other, while the complex_8:2 coating did not show significant differences relative to the other materials. The remaining mechanical property values indicate that the assessed films were generally similar.

Slightly different results were reported by [28], where the mechanical properties of single-layer films based on chitosan, furcellaran, and their complexes at ratios of 9:1, 8:2, and 7:3 were assessed. In that study, an increase in the furcellaran content led to a decrease in maximum breaking load, tensile strength, and elastic modulus, while elongation at break increased. Films prepared from the 7:3 complex showed the highest elasticity among the tested materials, which is consistent with the observations in this work. The introduction of chitosan was also noted to increase surface roughness, which may have contributed to the improvement of some mechanical properties. Similar trends were observed in [41], where mechanical properties were positively influenced when polymers were well mixed.

The thermal properties of the tested materials are presented in Table 3. The complex_8:2 film showed a slightly higher endothermic peak (Tpeak), which may indicate a trend toward increased thermal stability, although all results were statistically comparable (158–163 °C). The highest change in enthalpy (ΔH) was observed for the complex_9:1 film, with statistically significant differences, while the other films showed similar ΔH values. Despite the lack of differences in Tpeak values, indicating similar thermal stability of the tested samples, the highest enthalpy value for the film based on the 9:1 complex means that more energy must be supplied to destroy/melt this material, probably due to the formation of a larger number of crystalline structures. This confirms the lowest maximum elongation value observed for this sample (Table 3). In this case, the greater number of crystalline structures formed will reduce the material’s elasticity, causing it to break more quickly due to stretching. Differences in thermal characteristics could be attributed to interactions between polysaccharide functional groups, particularly between the -SO_3_^−^ group of furcellaran and the NH_3_^+^ cation of chitosan. In agreement with [28], increasing the furcellaran fraction in the complex can increase the heat released during thermal decomposition, as reflected in comparable ΔH results for mixtures at different ratios (9:1, 8:2, 7:3) as well as for pure furcellaran films. This parameter is related to the degree of crystallinity, which tends to rise with more intense crystallisation [42].

**Figure 7:**
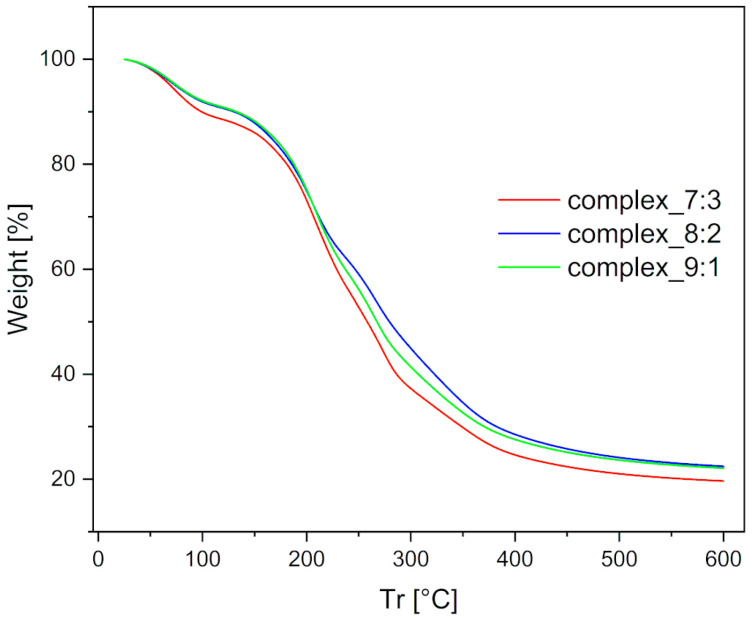
TGA profiles for the double-layer films.

The thermal stability of the prepared films was measured using the TGA method. The results revealed slight differences in mass variation on the TG curve, as shown in Figure 7.

The first mass loss was observed up to 130 °C and can be attributed to the removal of free or loosely bound water. The complex_7:3 film contained the highest amount of physically bound water (approximately 2.5% more than the others) and exhibited the most open structure, which suggests the presence of numerous hydrogen bonding interactions. The next stage of mass loss was observed in the range of 130–400 °C and was assigned to degradation caused by the breakdown of the complex, starting at around 170 °C. Among the tested films, the complex_7:3 film degraded the fastest and with the greatest mass loss. In contrast, the complex_8:2 film showed the lowest degradation intensity, indicating greater stability compared to the complex_9:1 film. This property was most likely due to interactions between the two oppositely charged polymers. Finally, the mass variation in the temperature range of 400–600 °C was associated with polymer degradation within the films. The total mass loss at 600 °C, related to dehydration, was within the experimental error and identical for all double-layer films.

## 3. Materials and Methods

### 3.1. Materials

Furcellaran (FUR) (Est-Agar AS, Kärla, Saare, Estonia)—type 7000, containing 79.61% polysaccharides, 1.18% protein and 0.24% fat, was used for this study. The molecular weight of furcellaran was 2.951 × 10^5^ g/mol. Chitosan (CH), with a molecular weight of 8.9 × 10^5^ g/mol, a degree of deacetylation of no less than 90% and a viscosity of 100–300 cP was purchased from POL-AURA (Zabrze, Poland). Glycerol (EUROCHEM BGD Sp z o.o., Tarnów, Poland) was used as a plasticiser. None of the chemical reagents was subjected to prior purification before use in the experiments.

### 3.2. Preparation of Double-Layer Films

The research material consisted of double-layer chitosan-furcellaran films. The first layer was a 1% furcellaran solution, while the second layer was a given chitosan and furcellaran complex at a ratio of 9:1, 8:2 or 7:3. The base layer was prepared by dissolving an appropriate amount of furcellaran in deionised water to prepare a 1% solution, which was then stirred at 70 °C for 24 h. The preparation of the top layer (9:1 ratio) began with the creation of individual biopolymer solutions. A 2% citric acid solution (PureLand) was prepared. Then, chitosan was dissolved in it to obtain a 1.8% solution. The suspension was stirred at room temperature for 24 h. In the case of the second biopolymer, a 0.2% furcellaran solution was prepared by dissolving it in deionised water. The mixture was stirred at 70 °C for 24 h. Chitosan–furcellaran complexes were formed by combining individually prepared polysaccharide solutions. For this purpose, the prepared solution was acidified with a 10% hydrochloric acid solution to a pH level of ~4. After that, it was brought to a temperature of 100 °C. When the set parameters were obtained, the hot furcellaran solution was added to the chitosan solution dropwise. Next, glycerine (1% m/m) was added to the prepared mixture and left to mix. Glycerin acts as a plasticiser in furcellaran–chitosan films, improving the elasticity and adhesion of the layers, but at the same time increases water vapour permeability and reduces the stiffness of the material [43,44]. The same procedure was used to produce chitosan-furcellaran complexes at the ratios of 8:2 and 7:3.

**Figure 8:**
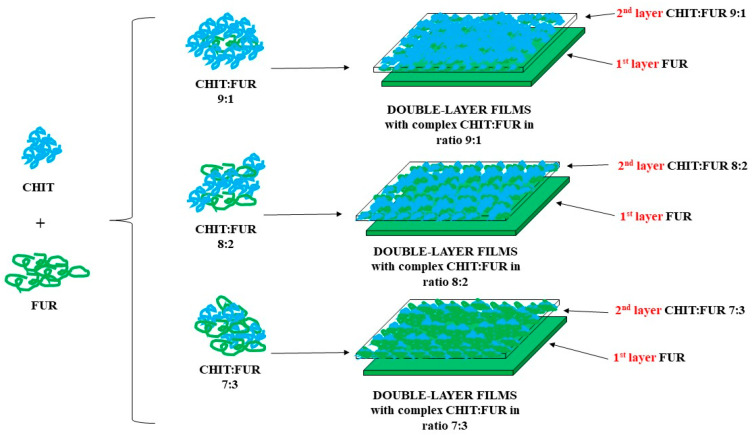
Scheme for obtaining double-layer films.

Double-layer films were made by pouring a base layer (1% furcellaran solution) onto plastic trays (food-grade PP trays (TILLGÅNG, IKEA, Älmhult, Sweden) were used, without additives or coatings). Before use, the trays were washed with distilled water, rinsed with alcohol (70% ethanol), and dried in the laboratory to reduce any surface contamination and volatile production residues. Then (after solidification), a chitosan–furcellaran complex at a given mass ratio. The whole complex was left to dry under a fume hood for 48 h and was later analysed. The procedure for obtaining the material for testing is presented in Figure 8.

### 3.3. Scanning Electron Microscopy (SEM)

Examination of the prepared films was carried out using the SEM/FIB Quanta 3D 200i (FEI) scanning electron microscope (FEI Company, Hillsboro, OR, USA), using a displacement voltage of 2 kV (current intensity of 0.25 nA) and a working distance of 10 mm. The samples did not require any prior preparation before imaging. Two locations (the most representative) were randomly selected from each type of tested film. Photographs of the sample surfaces were taken using magnifications of 150, 500, 800, and 1500 times, while cross-sections were imaged at magnifications of 200, 500, and 1000 times. The obtained results were presented in the form of photographs.

### 3.4. Atomic Force Microscopy (AFM)

The study was performed using a Dimension Icon XR atomic force microscope (Bruker, Santa Barbara, CA, USA). The device operates in air in the Peak Force Tapping (PFT) mode, using silicon cantilevers (Bruker) with a constant nominal elasticity of 0.4 N/m and a measuring tip radius of less than 10 nm. All tested films were cut into small pieces and then placed on a smooth silicon plate and imaged. Roughness parameters, RMS, were calculated using Nanoscope Analysis 1.9 software (Bruker) from 12 different areas of each sample.

### 3.5. Fourier Transform Infrared Spectroscopy

The absorption spectra of the tested films were measured using an FTIR spectrometer (Nicolet iS5, Thermo Scientific, Waltham, MA, USA). The FTIR spectra were recorded between 4000 and 700 cm^−1^ at a resolution of 4 cm^−1^. All spectra were recorded at room temperature (23 ± 0.5 °C).

### 3.6. Absorption Spectroscopy UV-VIS

The absorption and transmission spectra of the tested films were measured using a UV-VIS spectrophotometer (SHIMADZU, UV-2101PC, Kyoto, Japan). The analysis was performed within the wavelength range of 200–800 nm. All spectra were obtained at room temperature (23 ± 0.5 °C).

The quantitative index of light barrier was determined according to formula (Equation (1)) for three films and, for comparison, for chitosan and furcellaran:

(1) $$\mathrm{LBI}\;(\%)\;=(1-\frac{T_{VIS}}{T_{UV}})\;\times 100$$

LBI [%]—the quantitative index of light barrier;

T_VIS_—average transmittance in the Vis range (e.g., 400–800 nm);

T_UV_—average transmittance in the UV range (e.g., 200–400 nm).

### 3.7. X-Ray Diffraction (XRD)

The crystal structure of double-layer films was characterised by powder X-ray diffraction (XRD) using BRUKER D2 PHASER (Billerica, MA, USA). The Cu Kα radiation (λ = 0.154184 nm) in the range of 10–80° (2θ) with a step of 0.02° was used.

### 3.8. Film Thickness Measurement

The film thickness was measured using a Mitutoyo 7327 digital micrometre (Kawasaki, Kanagawa, Japan). Measurements were taken at 15 locations for each type of film, with an accuracy of 0.001 mm. The average value of this estimation was accepted as the film thickness.

### 3.9. Film Water Content and Solubility

Film water content and solubility were determined according to the proposed methods [45,46]. From the prepared films, three squares with a side length of 3 cm were cut out at random locations. Each square was weighed on an analytical scale to the nearest 0.0001 g (W_1_). Then, the film samples were placed in weighing bottles and dried in a dryer at 70 °C for 24 h (until a constant dry mass was obtained). After this time, the squares were cooled and then weighed on an analytical balance to the nearest 0.0001 g (W_2_). The dried samples were then immersed in 30 mL of deionised water and left to stand in it for 24 h at room temperature. After this time, the samples were removed from the water, placed in weighing bottles and dried again in a dryer at 70 °C for 24 h, and then weighed on an analytical scale to the nearest 0.0001 g (W_3_). The water content (W_c_) and solubility (R) of the film were calculated using the following equations (Equations (2) and (3)) [28]:

(2) $$W_{c}\;[\%]\;=\;\left(\frac{W_{1}-W_{2}}{W_{1}}\right)\times 100\%,$$

(3) $$R\;[\%]=\left(\frac{W_{2}-W_{3}}{W_{2}}\right)\times 100\%,$$

where

W_1_—baseline sample mass;

W_2_—sample weight after first drying;

W_3_—sample weight after second drying.

The analysis was performed in triplicate for each type of film.

### 3.10. Water Vapour Transmission Rate (WVTR)

Water vapour transmission rate testing was performed in accordance with the applicable standard [47]. A glass vessel was filled with silica gel, which was covered with the tested film. The samples prepared in this way were weighed on an analytical scale (W_1_) to the nearest 0.001 g and then placed in a microclimate chamber. They were stored for 24 h at 25 °C and 75% relative humidity. After this time, the samples were removed and weighed again (W_2_). The water vapour transmission rate (WVTR) was assessed, taking the increase in the sample mass into account, using the following formula (Equation (4)):

(4) $$WVTR\left[\frac{g}{m^{2}\times d}\right]=240\times \left(\frac{weight\;of\;water}{surface\;penetration}\right)\times 24$$

The measurement was performed in triplicate for each film type.

### 3.11. Water Contact Angle

The water contact angle is one of the most important properties when characterising the surface of a tested material. This measure is found between the tangent to the droplet surface (where it contacts the test sample) and the test sample surface. Measuring this angle provides information about the wettability of a solid surface.

The contact angle measurement was performed using the sessile drop method with the OCA 15EC optical goniometer (Data Physic Instruments GmbH, Filderstadt, Germany). A drop of deionised water, at a volume of 8 μm, was applied to the surface of the tested films using a microinjector, and the resulting image was recorded. The study was performed in ten replicates for each type of film, measuring the contact angle on both sides of the material (“top” and “bottom”) via SCA20 Software ver. 5.0.41 (Data Physic Instruments GmbH, Filderstadt, Germany).

### 3.12. Colour Measurement Parameters

Colour measurements were performed using the Color i5 spectrophotometer (X-Rite, Grand Rapids, MI, USA), using d/8 geometry. The spectral range was set to 400–700 nm. During the test, a D65 light source, an observation angle of 10° and a measuring slit of 25 mm were used. A white standard (white plate) was utilised as the background. Based on the obtained measurement data, the colour difference (ΔE*) of the tested films (L*, a*, b*) in relation to the applied background (L, a, b) was calculated according to the following formulas (Equation (5)), as well as the whiteness index (WI) (Equation (6)) adopting the assumption of L = 100:

(5) $$\Delta E=\sqrt{{\left(L-L^{*}\right)}^{2}+{\left(a-a^{*}\right)}^{2}+{\left(b-b^{*}\right)}^{2}}$$

(6) $$WI=100-\sqrt{{\left(100-L^{*}\right)}^{2}+{\left(a^{*}\right)}^{2}+{\left(b^{*}\right)}^{2}}$$

where

L, a, b—parameters of the white tile reference (background);

L*, a*, b*—parameters of the tested film.

Measurements of the L*, a*, b* parameters were performed in triplicate for each film type.

### 3.13. Mechanical Properties

The mechanical properties were determined in accordance with the standard [48] for plastics, which presents general principles of the procedure. The strength tests were carried out using the SHIMADZU EZ TEST EZ-LX device (Shimadzu Scientific Instruments, Kyoto, Japan). The test sample, rectangular in shape, was 300 mm long and 15 mm wide, and was subjected to a tensile force along the vertical axis, at a constant speed of 70 mm/min and at room temperature. The following mechanical properties were tested: maximum breaking load [N], tensile strength [kN/m], tensile index, maximum elongation [%], modulus of elasticity [N/mm^2^] and breaking length. Testing for a given parameter was performed in triplicate for each type of film.

### 3.14. Differential Scanning Calorimetry (DSC)

Assessment was performed using the DSC 4000 differential scanning calorimeter (PerkinElmer Inc., Springfield, IL, USA). The tested samples (approximately 5 mg each) were hermetically sealed in aluminium pans and then heated at temperatures from 30 °C to 250 °C, at a rate of 10 °C/min. An empty aluminium pan was used as a reference sample. The parameters (Tpeak and ΔH) were measured three times for each of the tested films. The temperatures and enthalpies of physical changes were determined via Pyris software ver. 13.3.30032 (PerkinElmer Inc., Springfield, IL, USA).

### 3.15. Thermogravimetric Analysis (TGA)

The thermal decomposition of double-layer films was studied by thermogravimetric (TG) analysis using a TGA/DSC 3+ (Mettler-Toledo, Columbus, OH, USA) instrument. The measurements were conducted at a temperature of 600 °C with a linear temperature increase of 10 °C/min. The TG runs were performed in an argon atmosphere.

### 3.16. Statistical Analysis

Statistical analysis of the obtained results was performed using STATISTICA 13.3 (StatSoft, Inc., Tulsa, OK, USA). One-way analysis of variance (ANOVA) with Tukey’s reasonable significant difference test (post hoc) was used at the significance level of *p* < 0.05 to determine the differences in the obtained results.

## 4. Conclusions

These double-layer films based on chitosan, furcellaran, and their complexes were successfully developed. The synthesis of CHIT–FUR complexes was confirmed via UV-VIS and FTIR spectroscopy. The results obtained using SEM and AFM confirmed the production of double-layer films, the layers of which differed from each other. The film with the highest proportion of chitosan to furcellaran (9:1) was characterised by the most uniform structure and surface. Based on the results of mechanical and physicochemical testing, it was found that the interactions in both the complex and the double-layer film are of an ionic and electrostatic type. The characterisation of the obtained double-layer films indicated that slightly better parameters were characterised by the film in which the ratio of chitosan to furcellaran was 9:1. For example, the films obtained in this way were characterised by the lowest water content, and this parameter increased with the increase in the share of furcellaran in the complex. In addition, the highest values of contact angle as well as water vapour transmission rate were observed in the double-layer films prepared in this manner. Depending on the CHIT–FUR ratio, the physicochemical, thermal, and mechanical properties of the double-layer films were different; however, these differences were not always statistically significant, which may contribute to increasing the range of their potential applications in food packaging. Despite interesting results, there are some limitations, such as high solubility and limited barrier performance, which are undesirable in traditional food packaging materials. However, these features may be beneficial, for example, in capsules, edible films and coatings, soluble sachets and active layers.

The high water vapour permeability (WVP/WVTR) renders the films “breathable”, which reduces condensation and may be advantageous for fresh products or wound dressings. At the same time, it decreases their ability to provide effective protection against moisture transmission (e.g., in the case of products sensitive to drying).

In the future, attempts should be made to introduce active ingredients or crosslinking agents into the films, designed in this way in order to improve the functionality and provide a reference for developing degradable food packaging. Further research is also demanded to evaluate the behaviour of these double-layer films when in contact with food matrices.

## Figures and Tables

**Table 1:** Roughness parameters RMS (arithmetic average height (Ra) and root mean square roughness (Rq)) for the double-layer films.

Film	Rq [nm]	Ra [nm]
complex_9:1 top	8.7 ± 0.8	6.6 ± 0.8
complex_9:1 bottom	29.4 ± 2.2	18.3 ± 2.5
complex_8:2 top	27.3 ± 4.6	20.4 ± 3.5
complex_8:2 bottom	25.3 ± 3.6	20.1 ± 2.9
complex_7:3 top	24.1 ± 3.2	15.3 ± 2.6
complex_7:3 bottom	28.0 ± 3.4	16.7 ± 2.8

**Table 2:** Film thickness, water content, solubility and contact angles of the prepared double-layer films (on both sides of the material, the “top” one—on the complex side and the “bottom” one—on the furcellaran layer side).

Film	Thickness [mm]	Water Content [%]	Solubility [%]	Top [°]	Image	Bottom [°]	Image
complex_9:1	0.209 ± 0.028	8.18 ^A^ ± 0.17	63.51 ^A^ ± 0.69	88.77 ± 1.18	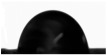	83.88 ± 1.51	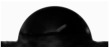
complex_8:2	0.202 ± 0.020	8.61 ^B^ ± 0.14	61.57 ^A^ ± 3.14	87.39 ± 0.66	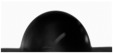	79.26 ± 2.39	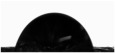
complex_7:3	0.203 ± 0.016	8.91 ^B^ ± 0.06	61.84 ^A^ ± 1.14	87.87 ± 0.66	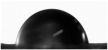	76.92 ± 1.92	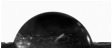

Values are the mean of three independent experiments ± standard deviation. Values marked with letters A and B indicate statistically significant differences for individual columns at a significance level of *p* < 0.05.

**Table 3:** Colour measurement of prepared double-layer films.

Film	L*	a*	b*	ΔE	WI ^c^
complex_9:1	89.18 ± 0.30	−0.15 ± 0.03	20.54 ± 0.52	18.63	76.78
complex_8:2	89.89 ± 0.26	−0.18 ± 0.03	18.12 ± 0.41	16.11	79.25
complex_7:3	89.97 ± 0.27	−0.05 ± 0.03	16.60 ± 0.35	14.66	80.60

Values are the mean of five independent experiments ± standard deviation. ^c^ WI—whiteness index.

**Table 4:** Mechanical and thermal property measurements of prepared double-layer films.

	Complex_9:1	Complex_8:2	Complex_7:3
Mechanical properties			
Maximum breaking load [N]	12.35 ^A^ ± 1.40	12.87 ^A^ ± 2.31	12.60 ^A^ ± 1.74
Tensile strength [kN/m]	0.82 ^A^ ± 0.09	0.86 ^A^ ± 0.15	0.84 ^A^ ± 0.12
Tensile index [-]	4.27 ^A^ ± 0.487	4.45 ^A^ ± 0.80	4.04 ^A^ ± 0.56
Maximum elongation [%]	21.24 ^A^ ± 0.61	25.29 ^A,B^ ± 1.98	28.48 ^B^ ± 3.41
Elastic modulus [N/mm^2^]	78.38 ^A^ ± 30.68	73.99 ^A^ ± 26.91	65.62 ^A^ ± 11.91
Breaking length [-]	0.43 ^A^ ± 0.05	0.45 ^A^ ± 0.08	0.41 ^A^ ± 0.057
Thermal properties			
Tpeak [°C]	158.1 ^A^ ± 5.6	162.9 ^A^ ± 6.2	160.4 ^A^ ± 4.9
ΔH [J/g]	190.17 ^B^ ± 9.4	146.96 ^A^ ± 4.5	145.19 ^A^ ± 5.4

Values are the mean of three independent experiments ± standard deviation. Values marked with letters A and B indicate statistically significant differences for individual rows at a significance level of *p* < 0.05.

## Data Availability

All data are contained within this article and are available upon request.
